# Prevalence and risk factors for placenta previa in a specialty hospital

**DOI:** 10.61622/rbgo/2025rbgo30

**Published:** 2025-07-15

**Authors:** Mara Elisa Monterde-Fernández, Joel Jahaziel Díaz-Vallejo, Iliana Rodríguez-Parissi, Berenice Venegas-Espinoza, Ezri Cruz-Perez

**Affiliations:** 1 Department of Obstetrics and Gynecology Centro de Alta Especialidad Dr. Rafael Lucio Xalapa Veracruz México Department of Obstetrics and Gynecology, Centro de Alta Especialidad Dr. Rafael Lucio, Xalapa, Veracruz, México.; 2 Teaching Department Centro de Alta Especialidad Dr. Rafael Lucio Xalapa Veracruz México Teaching Department, Centro de Alta Especialidad Dr. Rafael Lucio, Xalapa, Veracruz, México.; 3 Department of Obstetrics and Gynecology Centro de Alta Especialidad Dr. Rafael Lucio Xalapa Veracruz México Department of Obstetrics and Gynecology, Centro de Alta Especialidad Dr. Rafael Lucio, Xalapa, Veracruz, México.; 4 Department of Obstetrics and Gynecology Centro de Alta Especialidad Dr. Rafael Lucio Xalapa Veracruz México Department of Obstetrics and Gynecology, Centro de Alta Especialidad Dr. Rafael Lucio, Xalapa, Veracruz, México.; 5 Universidad Veracruzana Faculty of Chemistry-Pharmaceutical-Biological Xalapa Veracruz México Faculty of Chemistry-Pharmaceutical-Biological, Universidad Veracruzana, Xalapa, Veracruz.

**Keywords:** Placenta previa, Cesarean section, Placenta, Maternal age, Pregnant people, Risk factors

## Abstract

**Objective:**

Placenta previa is a risk factor for obstetric hemorrhage, which, if not managed, can lead to maternal and neonatal death. Most cases are diagnosed after 28 weeks of gestation; in many cases, prenatal diagnosis is not timely. The objective of this study was to estimate the prevalence of placenta previa and its risk factors.

**Methods:**

A retrospective case-control study was carried out with a total of 35 cases and 138 controls among pregnant women. The variables studied were previous cesarean section, BMI, abortions, uterine surgeries and maternal age, among others. The chi-square test was used to examine differences between groups; the OR was calculated for each factor via univariate and multivariate analyses.

**Results:**

The prevalence of placenta previa was 0.57%. The risk factors identified were advanced maternal age (OR 3.0; 95% CI 1.3-7.1) and previous cesarean section (OR 10.7; 95% CI 1.7-68.5).

**Conclusion:**

The prevalence of placenta previa was similar to that reported in the literature, and the most prevalent risk factors were advanced maternal age and previous cesarean section. The identification of risk factors in women with placenta previa makes it possible to establish action plans for personalized care during pregnancy and childbirth and to reduce complications.

## Introduction

Placenta previa is an obstetric complication and is due to its insertion in the lower uterine segment, either very close to or with some degree of coverage of the internal os of the cervix.^(1)^ Worldwide, the incidence is estimated to be between 0.3% and 2%.^(2)^ This condition is considered a risk factor for obstetric hemorrhage, which can lead to maternal and neonatal death secondary to the complications of postpartum hemorrhage.^(3)^

In recent decades, the incidence of placenta previa has increased, which is related to increases in cesarean section rates and maternal age.^(4,5)^ Its actual estimation is difficult because its evaluation and diagnosis require the skills of the attending physician and various diagnostic aids available in the hospital unit.^(6)^

According to the multidisciplinary consensus of several obstetric societies, if the placenta is less than 20 mm from the internal cervical os, but not covering, it is termed low lying, while any placenta covering the internal cervical os is classified as placenta previa, requiring cesarean delivery. The diagnosis is confirmed at 32 weeks gestation.^(7,8)^

In Mexico, the incidence of placenta previa varies between 0.33% and 2.6%.^(9,10)^ This condition has gained relevance because it is one of the main causes of obstetric hemorrhage, which, like the hypertensive state of pregnancy, is a cause of maternal death.^(11)^ The etiology of placenta previa is still unknown; however, it is related to several risk factors, such as previous abortion, multiparity, drug use, and advanced maternal age.^(12)^ An association has also been described with endometrial damage and alterations during uterine healing, which may be secondary to previous uterine surgical procedures, such as cesarean section or curettage, and even a history of placenta previa in previous pregnancies.^(13,14)^ In general, it is estimated that between 30% and 65% of cases occur in women with previous cesarean sections.^(6,15)^

In Mexico, the frequency of cesarean sections has been increasing; in 2012 alone, a rate of 45.58 per 100 births was recorded, and by 2021, it was expected to be 51.56 cesarean sections per 100 births.^(16)^ The excessive use of this practice exceeds the WHO recommendation of 15%.^(5)^ The timely identification of risk factors favors early surveillance and diagnosis of this pathology, as well as its adequate management according to clinical criteria. Therefore, the objective of this study was to estimate the prevalence of placenta previa and risk factors in pregnancies from 22-40 weeks of gestation at the Gynecology and Obstetrics Department of a specialty hospital in Xalapa, Veracruz, Mexico.

## Methods

**Figure 1:**
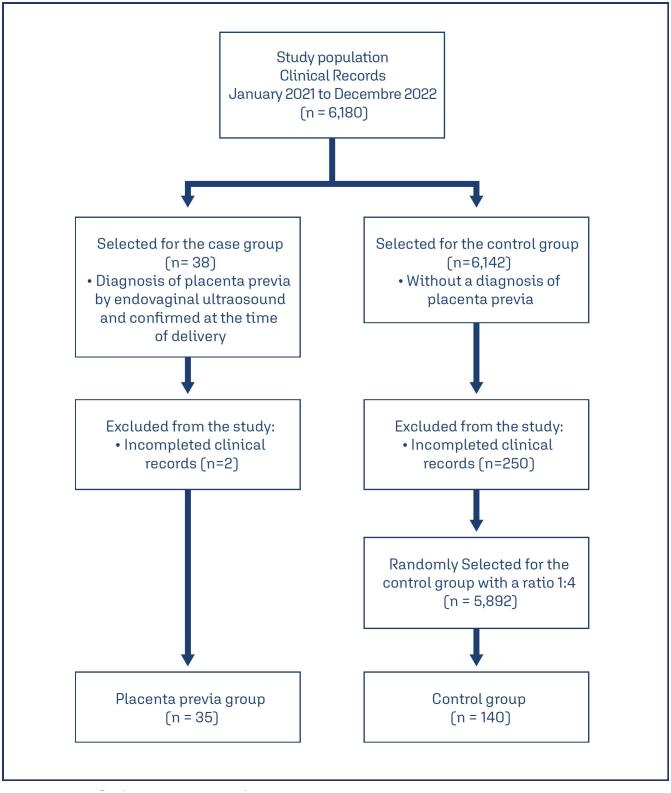
Selection of subjects

A retrospective case-control study was performed at a 1:4 ratio. The population consisted of patients who were pregnant at 22-40 weeks of gestation at the Gynecology and Obstetrics service of the Centro de Alta, especially Dr. Rafael Lucio, Xalapa, Veracruz, Mexico, from January 1, 2021, to December 31, 2022. The research was approved by the Hospital's Research Committee with registration number (COFEPRIS 19CI30087028) with folio CI 29/22. The information was collected from the clinical records; for the cases, the clinical records of patients with a diagnosis of placenta previa by endovaginal ultrasound and confirmed at the time of cesarean section were selected. The controls were records of patients who came for consultation during the same period but did not present a diagnosis of placenta previa. The controls were randomly selected patient records of women with vaginal or cesarean delivery attended in the same study period. 250 patients were excluded because of incomplete clinical records; for each case, 4 controls were randomly selected. The data were collected via an information collection sheet through the clinical records of the patients who met the inclusion criteria for both the case group and the control group, excluding those who had incomplete clinical records from both groups. 35 cases were included, whereas the control group consisted of 140 patients, with a total population of 175 subjects (Figure 1)

The variables analyzed were age, BMI, history of placenta previa, previous cesarean section, abortion, uterine surgery, drug use, maternal age, gestational age, comorbidities and prenatal care. These variables were selected according to risk factors that have been identified in previous studies. Normality tests were performed, and statistics according to their distribution were used. The prevalence of placenta previa was estimated, and an evaluation of the overall incidence of cesarean section, both the history of previous cesarean section and the resolution of the pregnancy was performed. Chi-square tests, as well as the Mann-Whitney U test, were used to evaluate the differences between the comparison groups. Bivariate and multivariate analyses were performed, as was a logistic regression model, to obtain odds ratios with their respective 95% confidence intervals. A p value of less than 0.05 was considered statistically significant.

## Results

Out of a total of 6,180 deliveries during the study period, 35 were cases of placenta previa. The calculated prevalence of placenta previa was 0.56% (95% CI 0.38–0.75%). The overall mean age was 29 years, with no difference between the group of women with placenta previa and the control group. The highest frequency of age was in the 25-29 years age group (26.6%), with respect to nutritional status; the overall BMI with the highest frequency was obesity (50.9%), followed by overweight (36.4%) and normal weight (12.7%). There was no difference between the cases and controls in terms of the frequencies presented (P=0.249). Among the main comorbidities found were gestational and type 2 diabetes mellitus (8.6%). The main clinical and sociodemographic characteristics of the cases and controls are presented in table 1.

**Table 1:** Comparison of the clinical and sociodemographic characteristics of the study population

Characteristics	Total n(%) (n=175)	Placenta previa n(%) (n=35)	Without placenta previa (n=140)	p-value
Age (Med, IQR)	29, 14	30.9, 13	28.5, 12	0.163
BMI (Med, IQR)	29.6, 5	30.2, 7	29.5, 6	0.538
Gravidity (Med, IQR)	2.6, 1	2.8, 2	2.5, 1	0.441
Parity (Med, IQR)	0.8, 2	0.7, 1	0.8, 2	0.287
Uterine surgeries (Med, IQR)	0.5, 1	0.7, 1	0.4, 1	0.015*
Abortions	39(22.3)	6(17.1)	33(23.6)	0.392
Cesarean sections	71(40.5)	22(62.8)	49(35.0)	0.002*
Prenatal care	154(88.0)	27(77.1)	127(90.7)	0.019*
Tobacco consumption	3(1.7)	0	3(2.1)	0.505
Comorbidities	142(81.1)	29(82.9)	113(80.7)	0.817

Med: median, IQR: interquartile range, Mann-Whitney U test, chi-square test, Fisher´s exact test

* p<0.05

Table 2 shows the risk factors for the presence of placenta previa. Advanced maternal age ≥ 35 years (OR 3.0; 95% CI 1.3–7.1), a history of cesarean section (OR 10.7; 95% CI 1.7–68.5) and poor prenatal care (OR 5.6; 95% CI 1.8–17.1) were associated with a greater chance of placenta previa. Parity and the presence of any comorbidity (diabetes mellitus, obesity or overweight) were not significantly associated with placenta previa.

**Table 2:** Logistic regression of risk factors for placenta previa

Variables		cOR (IC95%)	p-value	aOR (IC95%)	p-value
Age					
	<35 años	1		1	
	≥ 35 años	3.4 (1.6-7.5)	0.001*	3.0 (1.3-7.1)	0.010*
History of cesarean section					
	0	1		1	
	1	2.5 (1.1-5.7)	0.031*	10.7 (1.7-68.5)	0.012*
	>2	6.2 (2.0-18.8)	<0.001*	3.0 (0.9-9.9)	0.071
History of uterine surgeries					
	No	1		1	
	Yes	2.6 (1.2-5.6)	0.011*	1.3 (0.3-6.7)	0.728
Prenatal visits					
	≥5	1		1	
	<5	3.1 (1.2-8.3)	0.019*	5.6 (1.8-17.1)	0.003*
Comorbidity					
	No				
	Yes	1.1 (0.4-2.9)	0.817	1.2 (0.4-3.6)	0.738
Parity					
	0	1			
	1-2	1.22 (0.4-3.6)	0.715	0.3 (0.1-1.2)	0.077
	>3	1.66 (0.5-5.6)	0.413	0.6 (0.2-1.7)	0.597
Gravidity					
	1	1			
	2 a 4	1.26 (0.4-3.6)	0.665	0.78 (0.2-3.9)	0.772
	≥ 5	1.78 (0.4-7.3)	0.419	2.0 (0.6-6.7)	0.257

* P<0.05,

cOR= crude odds ratio and aOR= adjusted odds ratio: adjusted for maternal age, uterine surgery, cesarean section and prenatal care

In general, 63.6% of the pregnancies were resolved by cesarean section, and 36.4% were resolved by vaginal delivery. There was a significant difference (P<0.001) between the control group (54.3%) and the placenta previa group (100%). Among the patients with placenta previa, 62.9% presented with obstetric hemorrhage, 31.4% of whom required hemocomponents; among the controls, 5.1% presented with hemorrhage, and 1.4% required hemocomponents. Women with placenta previa were more likely to present with obstetric hemorrhage (OR 32.9; 95% CI 10.1-108.1) and the need for hemocomponents (Table 3).

**Table 3:** Logistic regression for maternal outcomes associated with placenta previa

Variables		cOR (IC95%)	p-value	aOR (IC95%)	p-value
Obstetric hemorrhage					
	No	1			
	Yes	24.3 (9.3-63.5)	<0.001	32.9 (10.1-108.1)	<0.001
Pregnancy resolution					
	Delivery	1			
	Cesarean	-	<0.001	-	-
Transfusion of hemocomponents					
	No	1			
	Yes	31.2(6.5-149.5)	<0.001	66.2 (10.7-407.9)	<0.001

* P<0.05,

cOR= crude odds ratio and aOR= adjusted odds ratio: adjusted for maternal age, uterine surgery, cesarean section and prenatal care

## Discussion

The prevalence of placenta previa was 0.57%, similar to the prevalence of 0.6% described by García-de la Torre et al.^(17)^ as well as that reported globally, which ranges from 0.3 to 2% and has become more evident as the prevalence of cesarean section has increased, as noted by Anderson-Bagga et al..^(2)^

In this study, a history of cesarean section was the most identifiable risk factor for the presence of placenta previa [OR: 10.7 (95% CI 1.7-68.5)], which is similar to that reported in other studies. Salim et al.^(18)^ conducted a study in Sudan Hospital and reported that 69.1% of patients with placenta previa had a history of cesarean section. Similarly, Kayem et al.,^(6)^ in a multicenter study in French hospitals, reported that a history of cesarean section is a risk factor for placental insertion anomalies and that the risk even increases as the number of previous cesarean sections increases. Gurol-Urganci et al.,^(19)^ in a UK study found that caesarean section at first delivery was associated with an increased risk of placenta previa for the second pregnancy. In contrast, the findings of this study are different from those reported by Senkoro et al.,^(12)^ who did not find an association with a history of cesarean section. Among the main risk factors they found was multigestation, whereas in this study, it was not an associated factor.^(12)^ In this study, the cesarean section rate was high at 40.5%, much higher than the WHO recommendation (15%) and higher than the global cesarean section rate of 21-29%.^(5)^ However, the rate reported for Latin America is 42.8%. Betran et al.^(20)^ reported that by 2030, there will be a global increase in the number of cesarean sections, and in the case of Latin America, this number could reach 54.3% .

Other risk factors for the presence of placenta previa in the population studied were maternal age equal to or greater than 35 years and the number of prenatal care visits, which contrasts with the findings of Senkoro et al.,^(12)^ who reported that maternal age equal to or greater than 35 years was associated with a lower likelihood of placenta previa. However, similar to the findings of this study, she reported that the presence of placenta previa was more common in women with fewer prenatal care visits, which could be because they were admitted earlier compared to their counterparts.

The findings of this study concerning maternal age and history of cesarean section are consistent with the findings of a meta-analysis by Jenabi et al.,^(21)^ who reported that advanced maternal age, abortion, cesarean section and tobacco use were considered risk factors for placenta previa. In the findings of this study, a history of abortion was not an associated factor, and tobacco use could not be evaluated since it was present in only the control group (2.2%).

In this study, we revealed an association between the presence of placenta previa and the likelihood of obstetric hemorrhage and, consequently, the need for blood components. Importantly, obstetric hemorrhage is one of the main causes of maternal death in this hospital. In this context, Roustaei et al.^(22)^ reported a greater prevalence of placenta previa in women aged 35 years and older, who are more likely to experience placental abruption and require blood transfusion.

More than 80% of the patients in this study presented with obesity and overweight; however, there was no difference between patients with placenta previa and those in the control group, although a high BMI is considered a risk factor for cesarean section, which could be related to the high rate of cesarean section that we found in this study.^(23)^ Importantly, all patients with placenta previa achieved a resolution of pregnancy via cesarean section; according to clinical practice guidelines, this resolution is an absolute indication for cesarean section; however, it is a constant in preserving the risk factors for future pregnancies.

A limitation of the study was the lack of inclusion of some variables that have been reported to be associated with placenta previa, such as fetal sex, drug use during pregnancy, and some fetal outcomes, such as the Apgar score, low birth weight and prematurity. This limitation of the study corresponds to the type of retrospective design, since it depends on the existence of a complete registry.

Finally, we determined that a history of cesarean section is strongly associated with the presence of placenta previa, which in turn can lead to complications during pregnancy and delivery, such as obstetric hemorrhage.

## Conclusion

The prevalence of placenta previa was similar to that reported in the literature, and the most prevalent risk factors were advanced maternal age and previous cesarean section. The specific identification of risk factors for placenta previa is useful in identifying women at risk of complications during pregnancy and childbirth to better follow up their pregnancies and thus provide comprehensive and personalized health care for mothers and babies.
